# Hepatitis Relapse after Yellow Fever Infection: Is There Another Wave?

**DOI:** 10.1590/0037-8682-0152-2020

**Published:** 2020-06-22

**Authors:** Fernanda Maria Farage Osório, Guilherme Grossi Lopes Cançado, Mateus Jorge Nardelli, Paula Vieira Teixeira Vidigal, Marcelo Antônio Pascoal Xavier, Wanessa Trindade Clemente

**Affiliations:** 1 Universidade Federal de Minas Gerais, Hospital das Clínicas, Instituto Alfa de Gastroenterologia, Belo Horizonte, MG, Brasil.; 2 Universidade Federal de Minas Gerais, Faculdade de Medicina, Belo Horizonte, MG, Brasil.; 3 Universidade Federal de Minas Gerais, Faculdade de Medicina, Departamento de Anatomia Patológica, Belo Horizonte, MG, Brasil.; 4 Universidade Federal de Minas Gerais, Faculdade de Medicina, Departamento de Propedêutica Complementar, Belo Horizonte, MG, Brasil.

**Keywords:** Yellow fever, Hepatitis, Dengue

## Abstract

During the yellow fever (YF) outbreak in Brazil, many cases of fulminant hepatitis were seen, although mild to moderate hepatitis was mostly observed with complete recovery. This report presents a case of late-onset hepatitis due to YF relapse. The patient sought medical attention after jaundice recurrence 40 days after the first YF hepatitis episode. This case highlights the importance of patient follow-up after the complete resolution of YF symptoms and discharge.

## INTRODUCTION

Yellow fever (YF) is an acute arboviral disease caused by a flavivirus transmitted in tropical areas of Africa and Latin America, with the possibility of determining urban cycles^1^. 

YF is described as a biphasic disease. The manifestation of the viremic phase is characterized by high fever, myalgia, and headache, usually lasting for around three days in mild and moderate cases. The toxemic phase occurs after the viremic period in 15% of patients. It is characterized by recrudescence of fever, worsening of the previous symptoms, and potential multiorgan damage, with a significant fatality rate^1^ ^,^ ^2^.

The urban transmission cycle of YF in Brazil was interrupted the 1940s due to wide vaccination coverage, but cases of the wild form have occurred sporadically in less populated regions of the country^1^. However, in December 2016, a large YF outbreak, which started in Minas Gerais, occurred in Brazil^3^.

Due to the outbreak magnitude, atypical YF manifestations were observed and studied. This report presents the case of a patient who sought medical attention due to hepatitis relapse after prior YF viral infection.

## CASE REPORT

A 42-year-old, otherwise healthy, male was admitted to the emergency room with a four-day history of malaise, myalgia, and high-grade fever. Laboratory tests showed increased liver enzymes (aspartate aminotransferase [AST] 2216 U/L and alanine aminotransferase [ALT] 1611 U/L), but normal bilirubin. Dengue Fever (DF) rapid test (NS1) and serology (ELISA IgM and IgG) were performed with negative results. Two days later the patient developed jaundice (total bilirubin 6.36 mg/dL and direct bilirubin 5.60 mg/dL) with a decrease in platelet count (52000 /mm^3^), normal international normalized ratio (INR) (0.85), and moderate proteinuria. At that time, abdominal ultrasonography showed hepatomegaly. Serology for viral hepatitis, Epstein-Barr virus, and Cytomegalovirus were negative. Dengue serology was repeated, and found positive, suggesting DF-related hepatitis. Despite an uncertain patient report of prior YF vaccination, YF polymerase chain reaction (YF-PCR) was performed considering the epidemiological risk and YF outbreak context. The patient was discharged after one week with improvement of laboratorial parameters (AST 259 U/L, ALT 638 U/L, alkaline phosphatase 153 U/L, platelets 294000 /mm^3^, and total bilirubin 2.25 mg/dL). The YF-PCR result was not available prior to discharge.

**FIGURE 1:**
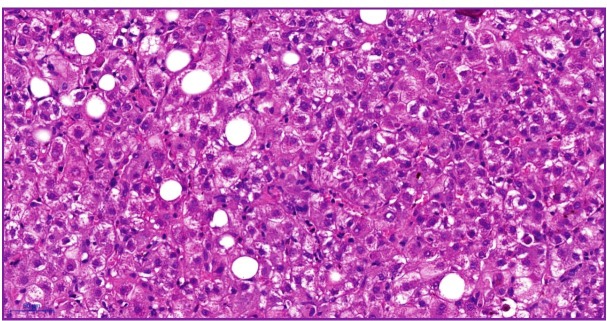
Few lobular inflammatory activity foci, some steatotic hepatocytes, and apoptotic bodies. Hematoxylin-eosin.

After 40 days, he was readmitted to the hospital with persistent fatigue, jaundice, and pruritus. Liver enzymes and bilirubin had increased (total bilirubin 19.2 mg/dL, AST 1037 U/L, ALT 2289 U/L, gamma-glutamyl transferase [GGT] 669 U/L, and INR 1.05). Extensive workup including serologies, ceruloplasmin, and ferritin was performed, but not indicative of a specific etiology. A hypothesis of autoimmune liver disorder was discarded by negative autoantibodies (*i.e.,* anti-nuclear, anti-smooth muscle, and anti-mitochondria antibodies) and normal serum gamma-globulin. Liver biopsy demonstrated lobular hepatitis with mild neutrophilic infiltration (Figure 1). The remaining parenchymal cells showed frequent hepatocyte ballooning, macro- and microvacuolar steatosis, and rare apoptotic bodies. The patient was discharged again after a small decrease in total bilirubin without a definitive diagnosis. A follow-up hepatology appointment was scheduled for five days later. 

Upon hepatologist consultation, it was found that the YF-PCR ordered during the first hepatitis episode was positive, thus corroborating the suspicion of relapsing hepatitis due to YF. A pathological review was performed, including YF virus immunohistochemistry stain, but it was negative. The patient was started on ursodeoxycholic acid (10 mg/kg) and within three days improved his pruritus and bilirubin levels. In the two-week follow-up period, liver enzymes were normal, and the medication was suspended.

## DISCUSSION

This report draws attention to the relapse of hepatitis caused by YF. The patient resides in Minas Gerais, an area of ongoing YF outbreak and the circulation of many other arboviruses. It is important to highlight that in endemic areas for arboviruses, a proportion of YF cases diagnosed by PCR present false positive results for DF serology, which could be related to previous antibodies or cross-reactivity^4^. The same phenomenon was recently reported in Singapore, where two patients with false positive results from rapid serological testing for DF were later confirmed to have severe acute respiratory coronavirus 2 infection causing COVID-19^5^. In our case, the uncertain history of prior YF vaccination caused a delay in the initial diagnosis. However, it is essential to mention that in Brazilian YF outbreaks, even some prior vaccinated patients have developed the disease, probably related to the reduction of neutralizing antibodies over time, breakdown of the vaccine cold chain, or even vaccine failure.

YF has a broad spectrum of clinical manifestations. Severe cases are characterized by hepatic dysfunction, renal failure, coagulopathy, and shock. Injury to hepatocytes has been described as involving the midzonal area of the hepatic lobule, cell death, Councilman bodies, and steatosis, as well as a discrete inflammatory infiltration^1^. During this recent YF outbreak, many cases of fulminant hepatitis were seen, including with liver transplantation treatment^6^, but most patients presented with mild hepatitis. 

Notably, other viruses can cause liver damage, and considering our epidemiological scenario in which various arboviruses circulate in the same geographical area, this might lead to a vastly differential diagnosis in the early phases of disease. Thus, some individuals with pre-existing immunity to heterologous flaviviruses develop broad cross-reactive antibody responses following infection with YF virus. Therefore, specific molecular tests should be performed for an accurate and timely diagnosis. In this report, although a positive DF serology was initially observed, subsequent RT-PCR^7^confirmed YF. 

Whereas the majority of liver damage from viral hepatitis occurs indirectly, related to a cellular immune response, it is believed that YF causes the direct cytopathic infection of hepatocytes leading to intense apoptosis. Indeed, Francis et al*.* previously demonstrated that histopathological features of continuing liver cell damage can be found up to two months after the initial infection, with the histology varying according to the stage of the disease^8^.

In this report, we present a case of relapsing hepatitis related to YF viral infection. After presenting typical hepatitis, the patient had partial resolution of clinical and biochemical indicators, followed by a severe self-limited relapse five weeks later. Relapsing hepatitis has been previously reported in picornavirus hepatitis A infection, in which persistent viremia and immune mechanisms responding to continuous antigenic stimulation were associated with a fluctuating disease course^9^.

Immunohistochemistry staining was performed using formalin-fixed and paraffin-embedded tissue that was collected at least 40 days after the onset of the symptoms. Although these results were negative, they did not refute the diagnosis. In fact, this might reflect a small viral load or that the hepatitis can be related to immune mechanisms and not exclusively to persistent viral replication.

To the best of our knowledge, this is only the fourth case of relapsing hepatitis secondary to YF^10^ ^,^ ^11^. The prior three cases were negative for YF-PCR serum assessment^10^. However, in one of the reports, YF antibodies were found, as well as histological positive YF-PCR and immunohistochemistry^11^. In our case, a histopathological assay with immunohistochemistry was performed; however, this showed negative result for virus detection. The fact that the liver tissue did not present YF virus during relapse hepatitis has not been demonstrated before, which could reinforce the role of an immune mechanism in relapsing hepatitis.

This case highlights the importance of following up with patients after the resolution of symptoms and discharge. Although a cholestatic pattern was evident, no sign of liver insufficiency was noted. Further studies may help to define the natural history of hepatic injury and how inappropriate immune responses may lead to relapsing or even fatal outcomes.
